# New pyrazolone derivatives, synthesis, characterization, and neuroprotective effect against PTZ- induced neuroinflammation in mice

**DOI:** 10.22038/IJBMS.2022.62869.13912

**Published:** 2022-12

**Authors:** Madiha Kanwal, Sadia Sarwar, Humaira Nadeem, Athar Ata, Fawad A. Shah, Sumra Malik, Saima Maqsood, Ghulam A. Miana

**Affiliations:** 1 Department of Pharmaceutical Chemistry, Riphah International University, Islamabad, Pakistan, 44000; 2 Department of Pharmacognosy, Riphah International University, Islamabad, Pakistan, 44000; 3 Department of Chemistry, University of Manitoba, Canada; 4 Department of Pharmacology, Riphah International University, Islamabad, Pakistan, 44000

**Keywords:** Anticonvulsant, Anti-inflammatory, Anti-Oxidant, Enzyme-linked - immunosorbent assay, Western blot, Pyrazolone

## Abstract

**Objective(s):**

The study was aimed at synthesis of the new derivatives of the pyrazolone nucleus, and their spectroscopic and pharmacological analysis and evaluation.

**Materials and Methods:**

Three series of compounds, with 2-picolinic acid (I a-d), 3-picolinic acid (II a-d), and 4-picolinic acid (III a-d) were synthesized and characterized by FT-IR, 1HNMR, 13C NMR, elemental, and melting points. The new compounds were then evaluated for their anti-oxidant, anti-inflammatory, and anti-epileptic potential. The hind paw edema model was used to screen anti-inflammatory potential, while the anticonvulsant effect was evaluated by employing the acute model of anti-epileptic activity. The *in vivo* anti-oxidant potential was determined through glutathione (GSH), glutathione-S-transferase (GST), catalase, and lipid peroxidase enzyme (LPO) assays. The expression of key biomarkers involved in inflammation and neuroprotection, including tumor necrotic factors alpha (TNF-α) and phosphorylated nuclear factor kappa B (NF-κB), was detected through enzyme-linked immunosorbent assay (ELISA) and Western blot analysis.

**Results:**

The tested compounds showed anti-oxidant potential. The selected compounds exhibited good anti-inflammatory potential. The PTZ-induced elevation of these inflammatory mediators and oxidative stress were ameliorated significantly by the selected compound Ic. Results of molecular analysis (ELISA and Western blot analysis) for potent compound Ic showed a prominent inhibitory effect against neuroinflammatory mediators, including TNF-α and NF-κB.

**Conclusion:**

It is concluded that the derivative Ic ameliorated PTZ-induced seizures, oxidative stress, and inflammatory cascades by regulating the NF-κB/ TNF-α/ROS pathway.

## Introduction

Five-membered heterocyclic compounds having two nitrogen atoms in their ring (diazole) possess a wide spectrum of pharmacological potential (1). Pyrazole is a five-membered ring containing two adjacent nitrogen atoms and three carbon atoms; the same ring with the keto (C = O) group may be called pyrazolone (2). This group represents bioactive molecules with a wide range of therapeutic potential and reported anti-inflammatory, antimicrobial, anti-tuberculosis, anti-viral, antidepressant, anti-cancer, and anti-convulsant actions (3). pyrazolone is one of the oldest classes of organic compounds with key significance in the pharmaceutical industry (4, 5). Antipyrine was first synthesized by Knorr in 1883 and was found to be biologically active as an antipyretic and analgesic compound (6). Though dipyrone and antipyrine are reported for rare incidences of agranulocytosis and blood dyscrasia yet keeping in view their therapeutic efficacy, the synthesis of new and safer derivatives is a continuously ongoing pursuit (7, 8). 

Epilepsy is associated with abnormal neuronal functioning of the epileptic brain and is a chronic disorder. Antiepileptic drugs (AEDs) protect against seizures but do not completely cure the disease. The outcome of treatment by AEDs depends upon the selection of the drug. Drug tolerance is the major determining factor towards AED selection rather than superior efficacy. Several AEDs drugs are available in the market for the treatment of epilepsy including carbamazepine, phenytoin, phenobarbital, and valproic acid clonazepam as major or alternative drugs (primidone, acetazolamide, etc.), depending upon the seizure classification. All AERDs are associated with depressed CNS function. Multiple issues associated with the use of these medications include drug interactions, addiction, tolerance, and severe side effects such as agranulocytosis, paresthesia, glaucoma, and teratogenicity (9). However, the mechanism of mediating epileptic disorder as well as the cause of drug resistance has grown substantially and created opportunities for the discovery and development of more efficacious anti-epileptic drugs (10). Therefore, the synthesis of new antiepileptic agents is urgently needed. 

Over the years multi-target therapeutic approaches have played a very positive role in drug development owing to the simultaneous interactions with multiple pathological targets (11). The relationship between epilepsy and inflammation is very obvious (12). Several key inflammatory mediators including TNF-α and NF-κB play a vital role in the pathophysiology of epilepsy (13). It is now evident that epileptic seizures are associated with the expression of elevated NF-κB and TNF-α levels in the neurons leading to the neuronal death and activation of glial cells which ultimately lead to pathological changes in the epileptic brain (14). The process of microglial activation is the main component of neuronal inflammation. This may be due to proinflammation, alteration in microglial morphology, up-regulation of cellular biomarkers such as calcium ionized calcium-binding adapter molecule 1(Iba-1), cannabinoid receptors 2 (CB-2), inducible nitric oxide synthetase (iNOS), cyclooxygenase 2(COX-2). Moreover, it is highly significant that CD14 ^+ ^monocyte-associated factors are linked with brain disorders and monocytes are primary producers of IL-6, IL-1β, and TNF-α (15). Pyrazolones possess good potential as anti-oxidant agents. Anti-oxidants inhibit or delay cellular damage by quenching free radical production (16-19). Oxidative stress is caused by the imbalance of two pathways, the extent of body anti-oxidants and the production of free radicals. The aging process speeds up by the excessive production of free radicals and results in oxidative stress. It may produce multiple body disorders like pain and inflammation (20, 21). 

**Scheme 1:**
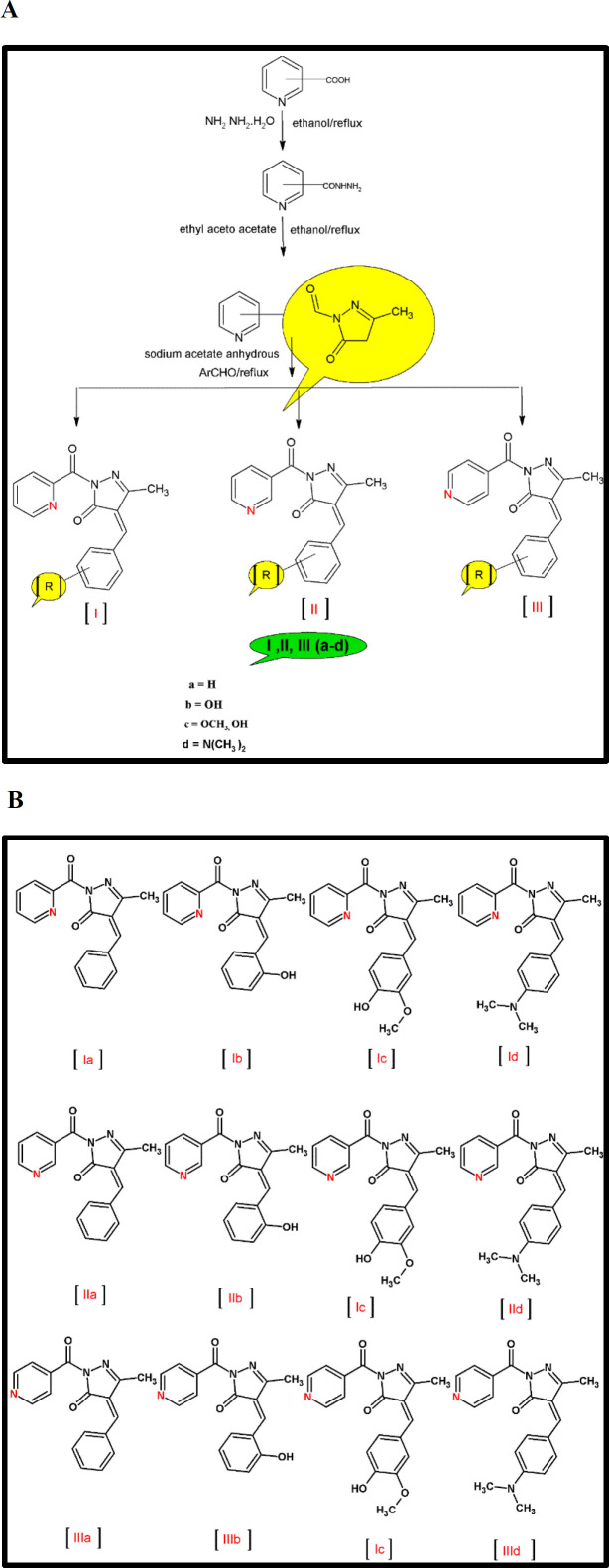
General Scheme for the synthesis of pyrazolone derivatives coupled with aryl aldehydes (A). Structures of all newly synthesized compounds (B)

We present in this article, the design, synthesis, docking, and pharmacological evaluation of a small library of different pyrazolones derivatives (Scheme 1), based upon the synthesis of pyrazolones from respective hydrazides and attachment of aryl aldehydes to get the final product. These derivatives were evaluated for anti-oxidant (DPPH assay), anti-inflammatory, and anticonvulsive potential by using the hind paw edema model. 

## Materials and Methods

***Chemicals and drugs***

The chemicals used were obtained from Sigma Aldrich (St. Louis, MO, USA). Melting points of all the new derivatives were recorded by the Digital Gallen Kamp apparatus (Sanyo, Osaka, Japan). A Bruker AM-300 (Billerica, Massachusetts, UK) was used to determine the proton NMR (^1^H NMR) spectra at 300 MHz. FT-IR spectrophotometer (ART eco ZnSe, Vmax in cm^-1^) was employed to determine the FT-IR of the synthesized compounds. Elemental analysis of synthesized compounds was determined by the LECO 183CHNS analyzer (Changsha, Hunan, China). All chemical reactions were monitored during the synthesis process with the help of Thin Layer Chromatography TLC. Hydrogen peroxide, formalin, 1-chloro-2,2-dinitrobenzene, glutathione (GSH), antibodies (mouse anti-TNF-α (CAT# E-EL-R0019) and mouse anti-NF-κB (CAT # E-EL-R0674) Avidin-Biotin complex (ABC) Elite Kit, and 3,3-diaminobenzidine peroxidase were purchased from Santa Cruz Biotechnology (Dallas TX, USA). Nicotinic acid, picolinic acid, isonicotinic acid, and aryl aldehydes were purchased from Sigma Aldrich (St. Louis, MO, USA). All reagents and chemicals were of 99% HPLC grade. 

***Animals***

Adult mice (20–25 g), 10–12 weeks old, were obtained from the Animal House at Riphah Institute of Pharmaceutical Sciences, Riphah International University (RIU), Islamabad, Pakistan. Every procedure was carried out according to the protocols approved by the Research and Ethical Committee (REC) at Riphah International University (Approval ID : Ref: No. REC/RIPS/2019/01). 

***General procedure for the synthesis of hydrazides from acid***

Equimolar (0.1 mol) of nicotinic acid/isonicotinic acid, picolinic acid, and hydrazine hydrate (0.1 mol) were mixed in a round bottom flask. In the reaction mixture, 80 ml of ethanol was added to dissolve the mixture. The reaction mixture was kept on reflux for 24 hr. Completion of the reaction was monitored with the help of TLC (3:1 and 1:3 ethyl acetate: pet ether). After the completion of the reaction, the solvent was evaporated by a rotary evaporator, and the solid obtained was recrystallized by ethanol. The pure form of hydrazides was confirmed by their melting points and R_f_ values. Their percentage composition was also calculated (22, 23).

*Pyridine-N-carbohydrazide*

IR: (Kbr) : cm^-1 ^3247.78 (N-H str), 3064.53 (C-H str- Ar-H), 2980.00 (C-H str- aliphatic), 1655 (C=O str), 1446 (C=C, str- Ar-H). ^1^HNMR: (DMSO d_6 _) δ ppm: 8.0 (s,1H, NH), 2.0 (s, 2H, NH _2 _). 7.35-9.15 (m, 4H, Ar-H). Yield 74%. m.p. 93-96 ^0^C. R_f _value 0.61. 

***General procedure for the synthesis of pyrazolone from hydrazides***

Equimolar quantities of Hydrazides (0.1 mol) and ethyl acetoacetate (0.1 mol) were taken and mixed in the ethanol. A few drops of Glacial acetic acid (GAA) were used as a catalyst. The reaction mixture was refluxed in a water bath for 10 hr. The reaction was monitored with the help of TLC (ethyl acetate: pet ether;1:3). After completion of the reaction, ethanol was separated from the reaction mixture and washed with ether to remove excess ethyl acetoacetate. The obtained product was dried, purified, and recrystallized with ethanol (24).

*2-nicotinoyl-5-methyl-2,4-dihydro-3H-pyrazole-3-one*

IR: (Kbr) : cm ^-1^ 3101 (CH str- Ar-H), 2948 (CH str, CH_3 _), 1654 (C=O str), 1600 cm^-1 ^(C=N str).H^1^ NMR: (DMSO-d_6 _): 7.4-7.95 (m, 4H, Ar-H), 2.29 (S, 2H, CH_2 _), 0.9(S, 3H, CH_3 _). Yield 75%. m.p. 118-120 ^0^C. R_f _value 0.81. Colorless needles.

***General procedure for the synthesis of 4-benzylidene-2-nicotinoyl-5-methyl-2, 4-dihydro-3H-pyrazole-3-one***

Pyrazolones of respective hydrazides (0.001 mole, 0.20g) were dissolved in a buffer solution of 10 ml glacial acetic acid and anhydrous sodium acetate (0.082 g, 0.001 mole) after which aryl aldehydes (0.106 g, 0.001 mole) were added into the above reaction mixture. The resultant mixture was refluxed for 12 hr, cooled, filtered, poured on crushed ice, and kept for some time. The solid obtained gradually appeared which was then filtered and dried. The solid was purified with the help of ethanol. The pure crystals were air-dried. 

*(4Z)-4-benzylidine)-5-methyl-2-(pyridine-2-ylcarbonyl)-2, 4-dihydro-3H-pyrazole-3-one. (Ia)*

IR (KBR): 3101 (C-H str, Ar-H), 2921 (C-H str, CH), 1709 (C=O str), 1592 cm^-1 ^(C=N str). H^1^ NMR: (DMSO-d_6, _δ ppm): 7.22-7.75 (m, 9H, Ar-H), 5.152 (s, 1H, =CH-Ar), 1.118 (s, 3H, CH_3 _). ^13^C NMR (DMSO d_6_, δ ppm): (19.1,1C), (102.2, 1C), (108.47, 1C), (119.02, 1C), (125.02,1C), (125.99, 1C), (137.01,1C), (138.25,1C), (138.77,1C), (148.23,1C), (149.54,1C), (154.53,1C), (156.31,1C), (160.78,1C), (164.14,1C), (172.80,1C), (180.24,1C). ( Anal. calculated for C _17_ H _13_ N _3_ O _2 _: C, 70.09%, H, 4.50%, N, 14.42%, O, 10.98%. M.W. 291.304. Yield 75%. m.p. 130-145 ^0^C. R_f _value 0.91. Colorless needles.

*(4Z)-4-(2-hydroxybenzylidene)-5-methyl-2-(pyridine-2-ylcarbonyl)-2,4-dihydro-3H-pyrazole-3-one (Ib)*

IR (KBR): 3101 (C-H str, Ar-H), 2922 (C-H str, CH), 1700 (C=O str), 1592 cm^-1 ^( C=N str). H^1^ NMR: (DMSO-d_6 _): 7.22-7.7 (m, 8H, Ar-H), 5.152 (s, 1H, =CH-Ar), 1.116 (s, 3H, CH_3 _), 9.20-9.23 (s, 1H, OH). ). ^13^C NMR (DMSO d_6_, δ ppm): (19.1,1C), (102.2, 1C), (108.47, 1C), (119.02, 1C), (125.02,1C), (125.99, 1C), (137.01,1C), (138.25,1C), (138.77,1C), (148.23,1C), (149.54,1C), (154.53,1C), (156.31,1C), (160.78,1C), (164.14,1C), (172.80,1C), (180.24,1C). Anal. calculated for C _17_ H _13_ N _3_ O _3 _: C, 66.44%, H, 4.26%, N, 13.67%, O, 15.62%. M.W. 307.303. Yield 65%. m.p. 125-135 ^0^C. R_f _value 0.71. Colorless needles.

*(4Z)-4-(4-hydroxy-3-methoxy benzylidine)-5-methyl-2-(pyridine-2-ylcarbonyl)-2,4-dihydro-3H-pyrazole-3-one (Ic)*

IR (KBR): 3100 (C-H str, Ar-H), 2922 (C-H str, CH), 1709 (C=O str), 1592 cm^-1 ^( C=N str). H^1^ NMR: (DMSO-d_6 _): 7.22-7.75 (m, 7H, Ar-H), 5.152 (s, 1H, =CH-Ar), 1.118 (s, 3H, CH_3 _), 9.23 (s,1H, OH), 3.9-4.06 (s, 3H, OCH_3 _). ). ^13^C NMR (DMSO d_6_, δ ppm): (19.1,1C), (24.13,1C), (102.2, 1C), (108.47, 1C), (119.02, 1C), (125.02,1C), (125.99, 1C), (137.01,1C), (138.25,1C), (138.77,1C), (148.23,1C), (149.54,1C), (154.53,1C), (156.31,1C), (160.78,1C), (164.14,1C), (172.80,1C), (180.24,1C). Anal. calculated for C _18_ H _15_ N _3_ O _4 _: C, 64.09%, H, 4.48%, N, 12.46%, O, 18.97%. M.W. 337.309. Yield 75%. m.p. 130-140 ^0^C. R_f _value 0.61. Colorless needles.

*(4Z)-4-(4-dimethylaminobenzylidene)-5-methyl-2-(pyridine-2-ylcarbonyl)-2,4-dihydro-3H-pyrazole-3-one (Id)*

IR (KBR): 3101 (C-H str, Ar-H), 2925 (C-H str, CH), 1709 (C=O str), 1592 cm^-1 ^(C=N str).H^1^ NMR: (DMSO-d_6 _): 7.22-7.74 (m, 8H, Ar-H), 5.142 (s, 1H, =CH-Ar), 1.116 (s, 3H, CH_3 _), 2.25 (s, 3H, CH _3 _). 2.504 (s,3H, CH_3 _). ^13^C NMR (DMSO d_6_, δ ppm): (11.65,1C), (19.1,1C), (24.13,1C), (102.2, 1C), (108.47, 1C), (119.02, 1C), (125.02,1C), (125.99, 1C), (137.01,1C), (138.25,1C), (138.77,1C), (148.23,1C), (149.54,1C), (154.53,1C), (156.31,1C), (160.78,1C), (164.14,1C), (172.80,1C), (180.24,1C). Anal. calculated for C _19_ H _18_ N _4_ O _2 _: C, 68.25%, H, 5.43%, N, 16.76%, O, 9.57%. M.W. 334.371. Yield 70%. m.p. 130-140 ^0^C. R_f _value 0.91. Colorless needles.

*(4Z)-4-benzylidine)-5-methyl-2-(pyridine-3-ylcarbonyl)-2,4-dihydro-3H-pyrazole-3-one (IIa)*

IR (KBR): 3101 (C-H str, Ar-H), 2921 (C-H str, CH), 1709 (C=O str), 1592 cm^-1 ^(C=N str).H^1^ NMR: (DMSO-d_6 _): 7.22-7.75 (m, 9H, Ar-H), 5.152 (s, 1H, =CH-Ar), 1.118 (s, 3H, CH_3 _). ). ^13^C NMR (DMSO d_6_, δ ppm): (19.1,1C), (102.2, 1C), (108.47, 1C), (119.02, 1C), (125.02,1C), (125.99, 1C), (137.01,1C), (138.25,1C), (138.77,1C), (148.23,1C), (149.54,1C), (154.53,1C), (156.31,1C), (160.78,1C), (164.14,1C), (172.80,1C), (180.24,1C). Anal. calculated for C _17_ H _13_ N _3_ O _2 _: C, 70.09%, H, 4.50%, N, 14.42%, O, 10.98%. M.W. 291.304. Yield 70%. m.p. 130-140 ^0^C. R_f _value 0.92. Colorless needles.

*(4Z)-4-(2-hydroxybenzylidene)-5-methyl-2-(pyridine-3-ylcarbonyl)-2,4-dihydro-3H-pyrazole-3-one (IIb)*

IR (KBR): 3101 (C-H str, Ar-H), 2922 (C-H str, CH), 1700 (C=O str), 1592 cm^-1 ^( C=N str).H^1^ NMR: (DMSO-d_6 _): 7.228-7.749 (m, 8H, Ar-H), 5.152 (s, 1H, =CH-Ar), 1.118 (s, 3H, CH_3 _), 9.204-9.232 (s, 1H, OH). ^13^C NMR (DMSO d_6_, δ ppm): (19.1,1C), (102.2, 1C), (108.47, 1C), (119.02, 1C), (125.02,1C), (125.99, 1C), (137.01,1C), (138.25,1C), (138.77,1C), (148.23,1C), (149.54,1C), (154.53,1C), (156.31,1C), (160.78,1C), (164.14,1C), (172.80,1C), (180.24,1C). Anal. calculated for C _17_ H _13_ N _3_ O _3 _: C, 66.44%, H, 4.26%, N, 13.67%, O, 15.62%. M.W. 307.303. Yield 64%. m.p. 125-131 ^0^C. R_f _value 0.74. Colorless needles.

*(4Z)-4-(4-hydroxy-3-methoxy benzylidine)-5-methyl-2-(pyridine-3-ylcarbonyl)-2,4-dihydro-3H-pyrazole-3-one (IIc)*

IR (KBR): 3100 (C-H str, Ar-H), 2922 (C-H str, CH), 1709 (C=O str), 1592 cm^-1 ^( C=N str).H^1^ NMR: (DMSO-d_6 _): 7.22-7.749 (m, 7H, Ar-H), 5.152 (s, 1H, =CH-Ar), 1.118 (s, 3H, CH_3 _), 9.232 (s,1H, OH), 3.9-4.06 (s, 3H, OCH_3 _). ^13^C NMR (DMSO d_6_, δ ppm): (19.1,1C), (24.13,1C), (102.2, 1C), (108.47, 1C), (119.02, 1C), (125.02,1C), (125.99, 1C), (137.01,1C), (138.25,1C), (138.77,1C), (148.23,1C), (149.54,1C), (154.53,1C), (156.31,1C), (160.78,1C), (164.14,1C), (172.80,1C), (180.24,1C). Anal. calculated for C _18_ H _15_ N _3_ O _4 _: C, 64.09%, H, 4.48%, N, 12.46%, O, 18.97%. M.W. 337.309. Yield 73%. m.p. 131-140 ^0^C. R_f _value 0.59. Colorless needles.

*(4Z)-4-(4-dimethylaminobenzylidene)-5-methyl-2-(pyridine-3-ylcarbonyl)-2,4-dihydro-3H-pyrazole-3-one (IId)*

IR (KBR): 3100 (C-H str, Ar-H), 2925 (C-H str, CH), 1708 (C=O str), 1591 cm^-1 ^(C=N str).H^1^ NMR: (DMSO-d_6 _): 7.22-7.74 (m, 8H, Ar-H), 5.142 (s, 1H, =CH-Ar), 1.116 (s, 3H, CH_3 _), 2.25(s, 3H, CH _3 _). 2.504 (s,3H, CH_3 _). ^13^C NMR (DMSO d_6_, δ ppm): (11.65,1C), (19.1,1C), (24.13,1C), (102.2, 1C), (108.47, 1C), (119.02, 1C), (125.02,1C), (125.99, 1C), (137.01,1C), (138.25,1C), (138.77,1C), (148.23,1C), (149.54,1C), (154.53,1C), (156.31,1C), (160.78,1C), (164.14,1C), (172.80,1C), (180.24,1C). Anal. calculated for C _19_ H _18_ N _4_ O _2 _: C, 68.25%, H, 5.43%, N, 16.76%, O, 9.57%. M.W. 334.371. Yield 78%. m.p. 130-142 ^0^C. R_f _value 0.93. Colorless needles.

*(4Z)-4-benzylidine)-5-methyl-2-(pyridine-4-ylcarbonyl)-2,4-dihydro-3H-pyrazole-3-one (IIIa)*

IR (KBR): 3101 (C-H str, Ar-H), 2921 (C-H str, CH), 1709 (C=O str), 1592 cm^-1 ^(C=N str).H^1^ NMR: (DMSO-d_6 _): 7.22-7.75 (m, 9H, Ar-H), 5.152 (s, 1H, =CH-Ar), 1.118 (s, 3H, CH_3 _). ). ^13^C NMR (DMSO d_6_, δ ppm): (19.1,1C), (102.2, 1C), (108.47, 1C), (119.02, 1C), (125.02,1C), (125.99, 1C), (137.01,1C), (138.25,1C), (138.77,1C), (148.23,1C), (149.54,1C), (154.53,1C), (156.31,1C), (160.78,1C), (164.14,1C), (172.80,1C), (180.24,1C). Anal. calculated for C _17_ H _13_ N _3_ O _2 _: C, 70.09%, H, 4.50%, N, 14.42%, O, 10.98%. M.W. 291.304. Yield 70%. m.p. 128-130 ^0^C. R_f _value 0.90. Colorless needles.

*(4Z)-4-(2-hydroxybenzylidene)-5-methyl-2-(pyridine-4-ylcarbonyl)-2,4-dihydro-3H-pyrazole-3-one (IIIb)*

IR (KBR): 3101 (C-H str, Ar-H), 2920 (C-H str, CH), 1700 (C=O str), 1592 cm^-1 ^( C=N str).H^1^ NMR: (DMSO-d_6 _): 7.228-7.749 (m, 8H, Ar-H), 5.152 (s, 1H, =CH-Ar), 1.118 (s, 3H, CH_3 _), 9.204-9.232 (s, 1H, OH). ^13^C NMR (DMSO d_6_, δ ppm): (19.1,1C), (102.2, 1C), (108.47, 1C), (119.02, 1C), (125.02,1C), (125.99, 1C), (137.01,1C), (138.25,1C), (138.77,1C), (148.23,1C), (149.54,1C), (154.53,1C), (156.31,1C), (160.78,1C), (164.14,1C), (172.80,1C), (180.24,1C). Anal. calculated for C _17_ H _13_ N _3_ O _3 _: C, 66.44%, H, 4.26%, N, 13.67%, O, 15.62%. M.W. 307.303. Yield 70%. m.p. 125-135 ^0^C. R_f _value 0.70. Colorless needles.

*(4Z)-4-(4-hydroxy-3-methoxybenzylidene)-5-methyl-2-(pyridine-4-ylcarbonyl)-2,4-dihydro-3H-pyrazole-3-one (IIIc)*

IR (KBR): 3102 (C-H str, Ar-H), 2920 (C-H str, CH), 1700 (C=O str), 1591 cm^-1 ^( C=N str).H^1^ NMR: (DMSO-d_6 _): 7.22-7.749 (m, 7H, Ar-H), 5.152 (s, 1H, =CH-Ar), 1.118 (s, 3H, CH_3 _), 9.232 (s,1H, OH), 3.9-4.06 (s, 3H, OCH_3 _). ^13^C NMR (DMSO d_6_, δ ppm): (19.1,1C), (24.13,1C), (102.2, 1C), (108.47, 1C), (119.02, 1C), (125.02,1C), (125.99, 1C), (137.01,1C), (138.25,1C), (138.77,1C), (148.23,1C), (149.54,1C), (154.53,1C), (156.31,1C), (160.78,1C), (164.14,1C), (172.80,1C), (180.24,1C). Anal. calculated for C _18_ H _15_ N _3_ O _4 _: C, 64.09%, H, 4.48%, N, 12.46%, O, 18.97%. M.W. 337.309. Yield 73%. m.p. 131-142 ^0^C. R_f _value 0.57. Colorless needles.

*(4Z)-4-(4-dimethylaminobenzylidene)-5-methyl-2-(pyridine-4-ylcarbonyl)-2,4-dihydro-3H-pyrazole-3-one (IIId)*

IR (KBR): 3100 (C-H str, Ar-H), 2925 (C-H str, CH), 1708 (C=O str), 1591 cm^-1 ^(C=N str).H^1^ NMR: (DMSO-d_6 _): 7.22-7.74 (m, 8H, Ar-H), 5.142 (s, 1H, =CH-Ar), 1.116 (s, 3H, CH_3 _), 2.25 (s, 3H, CH _3 _). 2.504 (s,3H, CH_3 _). ^13^C NMR (DMSO d_6_, δ ppm): (11.65,1C), (19.1,1C), (24.13,1C), (102.2, 1C), (108.47, 1C), (119.02, 1C), (125.02,1C), (125.99, 1C), (137.01,1C), (138.25,1C), (138.77,1C), (148.23,1C), (149.54,1C), (154.53,1C), (156.31,1C), (160.78,1C), (164.14,1C), (172.80,1C), (180.24,1C). Anal. calculated for C _19_ H _18_ N _4_ O _2 _: C, 68.25%, H, 5.43%, N, 16.76%, O, 9.57%. M.W. 334.371.Yield 77%. m.p. 130-141 ^0^C. R_f _value 0.92. Colorless needles.

***2, 2,diphenylpicrylhydrazide (DPPH) free radical scavenging activity***

Anti-oxidant activity of the newly synthesized compounds was performed through DPPH assay. Different concentrations (1 µg/ml, 3 µg/ml, 10 µg/ml, 100 µg/ml, 300 µg/ml, and 700 µg/ml) of the compounds were prepared. Initially, the stock solution of each synthesized compound (1 mg/10 ml) was prepared. 2 ml of the test sample was taken from each stock solution and 8 ml of DPPH [0.004%w/v] was added to it. All test tubes were kept in a dark area for at least 72 hr. The anti-oxidant potential was indicated qualitatively by observing the color change from violet to yellow. For qualitative data, the absorbance of each compound was measured by a UV Spectrophotometer at 517 nm. Ascorbic acid [vitamin C] was used as a reference standard; free radical scavenging activity of the compounds was calculated as given below (25).

$\mathrm{Percentage scavenging activity}=\frac{\mathrm{Absorbance of control}-\mathrm{Absorbance of sample}}{\mathrm{Absorbance of control}}\times 100$

***Anti-inflammatory activity***

Six compounds were selected for further *in vivo* anti-inflammatory potential. The anti-inflammatory activity of synthesized compounds was carried out by using the carrageenan-induced hind paw edema model in mice using 1.0% carrageenan solution via the subplanter route as a phlogistic agent. The animals were divided into 4 groups (n = 6) as follows: Group I, standard control which was administered with diclofenac sodium at 10 mg/kg; Group II, Vehicle Control administered with DMSO; Group III, Disease group: freshly prepared (0.05 ml, 1% w/v in 0.9% normal saline), carrageenan was injected by the oral route; Group IV, Treatment group administered with 10 mg/kg, 20 mg/kg, and 30 mg/kg of selected compounds. The volume of paw edema was measured by a water plethysmometer before and after 1, 3, and 5 hr of injection of carrageenan (26).

***Anti-epileptic activity***

All animals were divided randomly into 5 groups (n = 6) as follows: group I, vehicle control: (was injected with 0.9% saline and 2% DMSO via intraperitoneal route); group II, treatment group at the dose of 20 mg/kg of compound Ic was injected via intraperitoneal route 30 min before PTZ; group III, treatment group at the dose of 30 mg/kg of compound Ic was injected via intraperitoneal route, 30 min before PTZ; group IV; positive control (Diazepam group, was injected diazepam 30 min before PTZ via the intraperitoneal route to induce convulsion); group V, disease group (PTZ group, was administered PTZ 90 mg/kg via the intraperitoneal route (IP) to selected mice). The behavior of all animals was monitored for 30 min after the injection and latency to the first generalized myoclonic seizures, tonic-clonic seizures, as well as the duration of tonic-clonic seizures was measured and recorded. It was assessed by an unbiased experimenter. The behavior of all animals (27) was classified as stage 0: producing no response at all, stage 1: chewing, mouth jerks & facial twitching, stage 2: myoclonic seizures, stage 3: rearing & forelimb clonus, stage 4: tonic-clonic seizures and turning onto the side, stage 5: generalized clonic seizures and turning onto the back. The latencies to the onset of myoclonic and tonic-clonic seizures were noted for 30 min after the PTZ injection. Finally, all the animals were ethically euthanized by CO_2 _inhalation when the animals’ vital signs were lost. Every possible effort was made to reduce the animals’ suffering. 

***Enzyme-linked immunosorbent assays (elisa) for diagnosis of molecular markers***

NF-κB and TNF-α expressions were measured as per the manufacturer’s instructions by p-NF-kB kit (CAT # E-ELRO674 Shanghai Yuchun Biotechnologies, China) and TNF-α kit (E-EL-R0019 E lab science Biotechnology Inc, USA). The supernatant of brain tissues was stored at -80 °C and homogenized in a phosphate buffer solution containing protease inhibitors (Phenylmethylsulphonyl Fluoride PMSF). In the next step, the supernatant was collected after centrifugation (13,500X g for 1 hr) and was treated with designated antibodies in 96-well plates to quantify p-NF-kB and NFkB. The concentrations of these antibodies were calculated by using a microplate reader (BioTek ELx808, Biotek, Winooski, VT, USA). Calculated values were represented in the form of a picogram of cytokines per milliliter (pg/ml). The procedure was repeated three times (28). 

***In vivo anti-oxidant potential of active compounds***

*GSH and GST analysis*

The already reported method was used to quantify the level of GSH. Homogenized and diluted tissue was added to freshly prepared PBS solution and a solution of 5-5^’ ^–dithiobis (2-nitrobenzoic acid). The absorbance of the solution was measured at 412 nm wavelength. The concentration of GSH S-transferase was measured by the given method with slight modification. Concisely the same concentration of GST and 1, chloro2, 4-dinitrobenzene were mixed and diluted with 0.1M solution of PBS (pH 6.5). Serial dilution was made, and absorbance was measured at 340 nm (29).

*Lipid peroxidation assay (LPO)*

Lipid peroxidation assay (LPO) was performed as per the reported procedure with few modifications (30). Lipid peroxide level was estimated by measuring the malondialdehyde (MDA) level in the hippocampus and cortex of brain homogenates of respective mice.

*Catalase assay*

Catalase assay was performed as per the reported method mentioned before with slight changes (31). 10 µl of the sample was added to each well followed by the addition of 290 µl of 3% of H_2 _O_2. _This 96-well plate was stored at room temperature in darkness for at least 10 min and absorbance was measured at 440 nm wavelength.

***Western blot analysis***

Western blot analysis was performed following the reported protocol with slight changes (32). For this purpose, hippocampus (dentate gyrus) and cortex from all treated mice were lysed in buffer solution and then homogenized. Bicinchoninic acid (BCA) protein assay kit was used to find out protein contents. Protein homogenate of 30 µg was loaded into sodium dodecyl sulfate-polyacrylamide gel electrophoresis which was afterward transferred into a polyvinylidene fluoride membrane. These membranes were incubated overnight at 4 °C with primary anti-TNF-α and anti-NF-κB antibodies, then blocked with bovine serum albumin (5%) at room temperature for 1 hr. These were washed in triplicate using 0.1% tween 20 with tris-buffered saline. These membranes were further reacted with secondary antibodies such as goat serum and anti-rabbit with 1:1000 dilution at room temperature for 90 min. An upgraded western blotting substrate kit was used for the visualization of immunoreactive bands. The evaluation and quantification of protein expression were carried out by using densitometry ImageJ software. 

***Statistical analysis***

Data was calculated in the form of mean ± SEM (n=6). One-way analysis of variance (ANOVA) was applied to analyze the data followed by *post hoc* Bonferroni multiple comparisons using Graph Pad Prism-6 (San Diego, CA, USA). Symbol *** or ### shows significant difference at *P*<0.001, while * and ** show significant differences at *P*<0.05 and *P*<0.01, respectively. The symbol * represents the significant difference relative to the control group and the symbol # shows the significant difference relative to the disease group.

## Results

***Anti-oxidant effect of pyrazolone derivatives***

**Figure 1:**
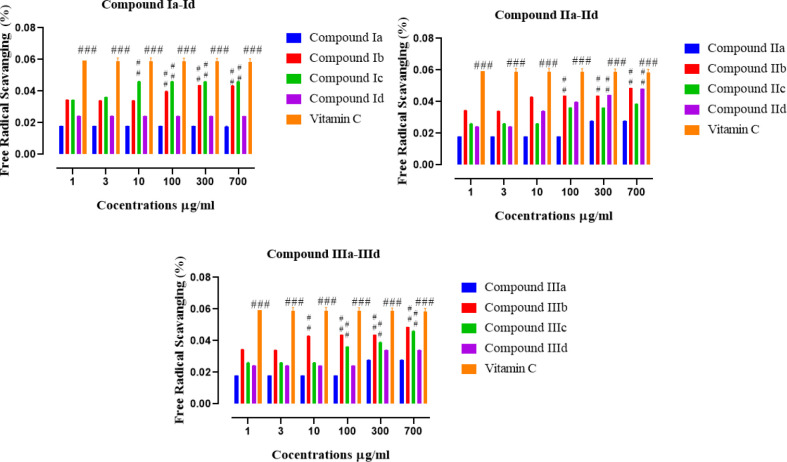
Illustrates the percent free radical scavenging of all newly synthesized compounds (Ia-Id), (IIa-IId), (IIIa-IIId), and vitamin C. Values are expressed as Mean ±SEM. Symbol *** or ### shows significant difference at *P*<0.001, while * and ** show significant differences at *P*<0.05 and *P*<0.01, respectively

All the synthesized compounds were initially screened for their anti-oxidant potential *in vitro* through a DPPH assay in terms of free radical scavenging activity. The results of compounds belonging to three series are shown in Figure 1A-C. The compounds Ib and Ic were potent anti-oxidant agents in series I; IIb and IId in series II, while IIIb and IIIc were in series III (Figure 1A, B & C). Only these compounds were used for further pharmacological activities. 

***Evaluation of the selected compounds for anti-inflammatory effect***

**Figure 2:**
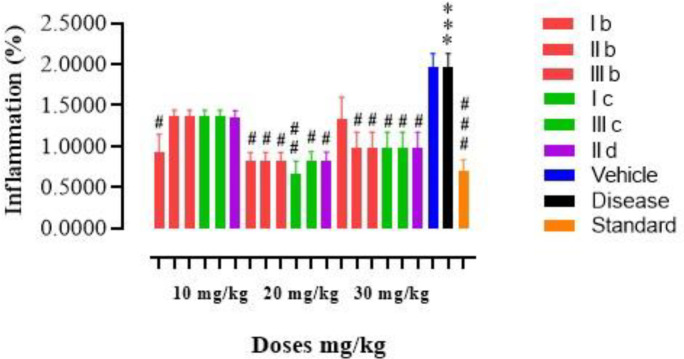
Anti-inflammatory effects of tested compounds Ib & Ic, IIb & IId, and IIIb & IIIc using the hind paw edema model on mice. Results were compared with standard anti-inflammatory drugs (diclofenac sodium). Data were presented in the terms of standard error Mean±SEM (n = 6). *P*˂0.05 was considered statistically significant. Symbol *** or ### shows significant difference at *P*<0.001, while * and ** show significant differences at *P*<0.05 and *P*<0.01, respectively. The symbol * represents the significant difference relative to the control group and symbol # shows the significant difference relative to the disease group

The anti-inflammatory potential was evaluated through carrageenan-induced paw in experimental mice. Edema is presented as a percentage increase in the hind paw as compared with the un-injected hind paw. Percentage change (% age) in paw volume was calculated and expressed as inflammation. The compounds (Ib, Ic, IIb, IId, IIIb, and IIIc) were administered to the mice at three different doses (10, 20, and 30 mg/kg) to observe the anti-inflammatory response. The results revealed that compound Ic at 20 mg/kg exhibited the best anti-inflammatory effect. All results were expressed in the form of graphs in Figure 2.

**Antiepileptic effect of the compound Ic**

**Figure 3:**
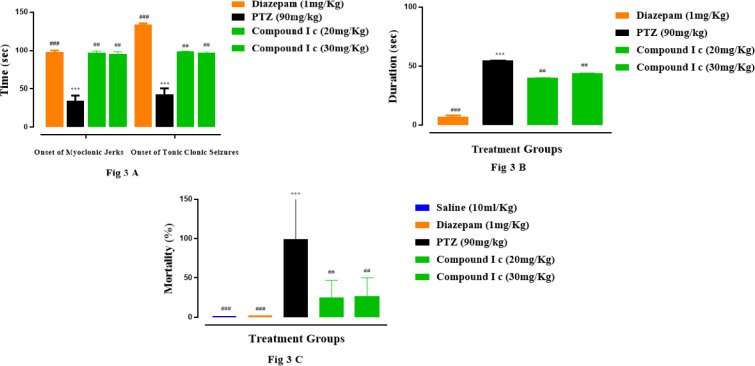
Effect of compound Ic, diazepam on the onset of tonic-clonic and myoclonic seizures and duration of tonic-clonic seizures and mortality rate. Symbol *** or ### shows significant difference at *P*<0.001, while * and ** show significant differences at *P*<0.05 and *P*<0.01, respectively. The symbol * represents the significant difference relative to the control group and symbol # shows the significant difference relative to the disease group. Data were expressed as Mean ± SEM (n = 6)

Compound Ic was selected based on its best anti-inflammatory potential for further study. It delayed the onset of PTZ (90 mg/kg) mediated myoclonic jerks, and tonic-clonic seizures as well as the duration of tonic-clonic seizures and inhibited the mortality rate, Figures 3 A, 3 B & 3 C, respectively. The compound Ic delayed the time of onset of myoclonic (38.10±2.6 in case of PTZ) and tonic-clonic seizures (45.7 ±8 in case of PTZ) to 80.15±2.5 and 85.14±1.43 sec, respectively at 20 mg/kg (*P*<0.01). At a further, higher dose, i.e., 30 mg/kg the response was even better with a further increase in the time of onset of myoclonic (78.15±2.1) and tonic-clonic jerks (80.15±1.42) while the duration of jerks was also reduced further to 45 sec from 55 in case of PTZ (Figures 3 A & 3 B). The PTZ group showed 100% mortality (*P*<0.05) while compound Ic reduced the mortality rate up to 25% (*P*<0.01 vs saline). Diazepam (1 mg/kg) exhibited 0% mortality (*P*<0.001 vs saline group) (Figure 3 C).

***Anti-oxidant effect of compound (Ic) on oxidative enzyme variation***

The anti-oxidant potential of compound Ic is evident in Table 1. Ethanol induced the accumulation of ROS while decreasing the levels of anti-oxidant enzymes (catalase, GST, and GSH) in the brain (*P*˂0.001). Administration of compound Ic remarkably ameliorated the down-regulated anti-oxidant enzymes as in Table 1.

***Effect of compound Ic on PTZ-induced lipid peroxidation (LPO)***

Several studies revealed malondialdehyde (MDA) as a fundamental indicator to determine oxidative stress. Thiobarbituric acid reactive substances (TBRAS) is a widely used procedure to determine lipid peroxidation product malonaldehyde (MDA). The test was performed as per the reported protocol. After the administration of ethanol, there was a valuable increase in lipid peroxidases, which were reduced when treated with compound Ic. The data obtained are shown in Table 1. Compound Ic prominently down-regulated the LPO level.

***Effect of Compound Ic on PTZ-induced neuroinflammatory mediators as detected through ELISA***

**Figure 4:**
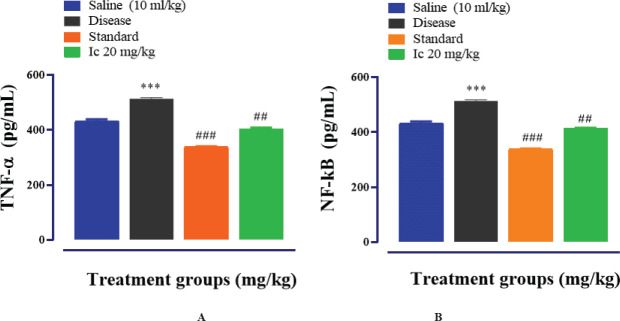
Represents the protein expression of TNF-α (A) and NF-κB (B). Compound Ic revamps the release of inflammatory mediators, expressed by ELISA. The data are expressed as Mean ± SEM. n = 6/group. Symbol *** or ### shows significant difference at *P*<0.001, while * and ** show significant differences at *P*<0.05 and *P*<0.01, respectively. The symbol * represents the significant difference relative to the control group and the symbol # shows the significant difference relative to the disease group

The brain homogenate from the above experiment was used for detecting the levels of selected key inflammatory markers, including NF-κB and TNF- α through ELISA. The expression of these markers was raised in PTZ-treated mice while compound Ic significantly reduced these levels in mice (Figure 4 A& B) (*P*<0.01). 

***Effect of compound Ic on PTZ-induced neuroinflammatory mediators as detected through Western blot analysis***

**Figure 5.**
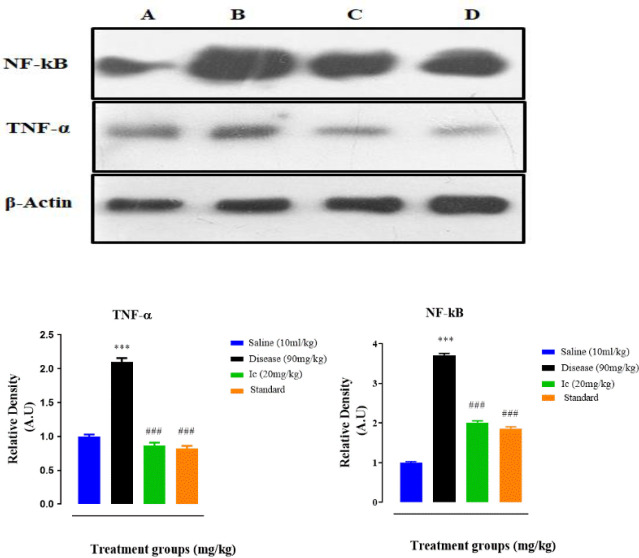
Inhibitory effect of treated compound (Ic) on tumor necrotic factor alpha (TNF-α) and phosphorylated -nuclear factor kappa B (NF-κB) expression in brain tissues implementing western blot analysis (above). Below is the graphical expression of data analyzed through One-way Anova and post hoc Turkey s’ test. All values were expressed as Mean ±SEM (n = 6). Symbol *** or ### shows significant difference at P<0.001, while * and ** show significant differences at *P*<0.05 and *P*<0.01, respectively. The symbol * represents the significant difference relative to the control group and the symbol # shows the significant difference relative to the disease group

The tissues of the hippocampus and cortex of the brains of treated mice in the case of control (A), PTZ-induced (B), test compound Ic (C), and standard drug (D) were subjected to western blot analysis. It is clear that the compound Ic revamped these levels in treated mice (Figure 5). Bands for compound Ic, diseased group (PTZ-induced), saline, and standard (β-actin) represent their effect and there is a remarkable reduction in the expression of NF-κB and TNF-α in the case of compound Ic as compared with the diseased group. 

**Table 1:** Effect of compound Ic on anti-oxidant enzyme and lipid peroxidation

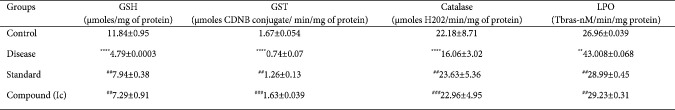

Symbol *** or ### shows significant difference at *P*<0.001, while * and ** show significant differences at *P*<0.05 and *P*<0.01, respectively. The symbol * represents the significant difference relative to the control group and the symbol # shows the significant difference relative to the disease group. All the above data are shown in the form of Mean ±SEM (n = 6)

GSH: Glutathione; GST: Glutathione-S-transferase; TBARS: Thiobarbituric acid reactive substances; LPO: Lipid peroxidation; CAT: Catalase

## Discussion

New derivatives of the pyrazolone nucleus were synthesized, characterized, and evaluated by spectroscopic analysis including FT-IR (with a prominent peak at 2922 (C-H str, CH)),^1^HNMR, and ^13^C NMR. Elemental analysis, M.W, M.P, and R_f _values were also carried out. Preliminary screening of all the new derivatives was done by *in vitro* DPPH free radical assay, to avoid unnecessary loss of animals. Among the tested compounds, 6 derivatives (Ib, Ic, IIb, IId, IIIb, and IIIc) exhibited remarkable anti-oxidant potential which were selected for further *in vivo* analysis (Figure 1A-C). Many established reports revealed that pyrazolone derivatives exhibited not only endogenous anti-oxidant and free radical scavenging effects (33-35), but also activate many anti-oxidant systems yielding species with unpaired electrons delocalized in the heterocyclic ring (36). The ability of these derivatives as anti-oxidant agents may be attributed to their proton donor ability to neutralize the free radicals. Results of *in vivo* anti-inflammatory assay employing the paw edema model showed a significant anti-inflammatory effect in the case of a few compounds (Figure 2). The compound (Ic) exhibited the best neuroprotective effect (Figure 3). Neuroinflammation is directly related to ROS-generated oxidative stress which further aggravates the already done loss. Several neuroinflammatory cytokines and mediators (COX-2), inducible nitric oxide synthase (iNOS), TNF-α, and IL-10. IL-6, mitogen-activated protein kinase (MAPKs), nuclear factor-kB, and (NF-κB) activation is reportedly triggered by neuroinflammation (37, 38). 

We selected only the Ic compound for further investigating its neuroprotective effect in *in vivo* model of epilepsy. The anticonvulsant effect was determined in terms of the onset of myoclonic and tonic-clonic seizures and the duration of tonic-clonic seizures. The compound Ic showed a significant anticonvulsant effect in terms of delay of both types of seizures, duration and mortality rate; the effect was close to that of the standard drug diazepam (Figures 3A, 3B & 3C). NF-κB is activated in neurodegenerative events including epilepsy. PTZ-induced activated NF-κB ultimately elicits the production of pro-inflammatory factors, such as TNF-α, IL-1β, and nitric oxide, further aggravating the damage. Both COX-2 and iNOS are toxic mediators of the inflammatory cascade which can be down-regulated by inhibiting NF-κB. The higher expression of NF-κB and TNF-α in our study was down-regulated in the brain of animals as detected through ELISA and Western blot analysis (Figures 4 & 5). In accordance with other results, anti-oxidant activity was also significant (Table 1). The reduced expression of GST and GSH was significantly restored by compound Ic. 

## Conclusion

PTZ-induced seizures activated inflammatory mediators including NF-κB and TNF-α along with ROS-mediated oxidative stress. The derivative Ic ameliorated PTZ-induced seizures, oxidative stress, and inflammatory cascades through NF-κB/ TNF-α/ROS pathway.

## Ethical Approval

During experimentation, every procedure was carried out according to the protocols approved by the Research and Ethical Committee (REC) at Riphah International University, Pakistan (Approval ID: Ref: No. REC/RIPS/2019/01).

## Authors’ Contributions

SS and HN Provided the concept; MK, SM, and SM Performed data curation; FAS, HN, and SSHA did formal analysis; AASS, HN, FAS, HAMK, SM, and SM were responsible for funding acquisition; SS and HN Provided methodology; SM Worked with software; MK Wrote the original draft; SS and IM Reviewed and edited the draft; All authors mutually consent to publish this data.

## Conflicts of Interest

The authors have no conflicts of interest.
